# Effects of the European hornet (*Vespa crabro* Linnaeus 1761) crude venom on its own species

**DOI:** 10.1186/1678-9199-19-4

**Published:** 2013-03-18

**Authors:** Jerzy Nadolski

**Affiliations:** 1 Faculty of Biology and Environmental Protection, Natural History Museum, University of Łódź, Kilińskiego 101, Łódź, 90-011, Poland

**Keywords:** European hornet, Vespa crabro, Hornet venoms, LD_50_

## Abstract

**Background:**

Lethal dose 50% is a classical index of toxicity that usually employs small rodents as experimental animals. Therefore, scarce data are available on the effects of venom on invertebrates, particularly the impact of wasp venom on its own species.

**Findings:**

In the present study, the lethality of *Vespa crabro* venom on its own species was studied. Lethal dose 50% values of crude venom on workers of hornet *Vespa crabro* were estimated to be 4.0 mg/kg of body weight.

**Conclusions:**

Wasps can use their venom apparatus effectively when attacking foreign workers that appear in the immediate vicinity of their nest. The toxins released during stinging are potent enough to kill. The result of this study eliminates the popular myth that venomous animals can be resistant to their own venom.

## Findings

The classical method of determining the toxicity of a substance is the lethal dose 50% (LD_50_), which is often used in analysis of different animal toxins [1-6]. Almost all studies about venom activity are based on the lethality of small rodents including mice and rats. There is little published data on the effects of venom on invertebrates [7]. Only a few studies on the lethal activity of a venom on its own species demonstrated significant results [8]. In addition, the degree of toxicity of venoms on individuals of their own species is unknown.

In the present study the toxicity of the European hornet *Vespa crabro* (Linnaeus, 1758) venom in relation to workers of its own species was assessed. Based on the author’s own personal observations, it can be stated that hornets also sting to defend their nest against intruders of their own species, but from alien colonies. Thus, this study attempted, for the first time, to answer the following questions: did natural selection create defense mechanisms to protect these insects against their own toxins and how hornets are sensitive to their own venom?

The analysis of the toxic activity of *Vespa crabro* venom was carried out on workers from two colonies of hornets established in Łódź city in Central Poland. Hornet venom was obtained by irritating insects with tweezers on their torso and abdomen, resulting in stinging reaction. The secreted venom was collected on watch glasses [8]. Then, frozen dried venom was stored in the dark at −20°C until used. In order to obtain percent values of dry matter from liquid venom, specified quantities of venom were weighed [6]. The final venom concentration was adjusted with PBS (137 mM NaCl, 10 mM phosphate, 2.7 mM KCl, pH 7.4) [7].

After weighing, each hornet worker received, into the abdomen, the appropriate amount of venom by using a 1 μL Hamilton microsyringe (USA). LD_50_ values for hornet workers (twenty workers per dose) at 24 hours were determined by the standard statistic method based on probit analysis [9-14]. Controls consisted o hornets injected only with PBS.

Table 1 displays the toxicity of *Vespa crabro* venom assessed on representatives of its own species. The obtained results underwent statistical analysis, including probit transformation, and the final value of LD_50_ is presented in Table 2.

**Table 1:** **Analysis of probit-transformed mortality of European hornet*Vespa crabro*workers provoked by their own venom**

**Dose (mg/kg of body weight)**	**Log dose**	**Actual mortality (%)**	**Probit-transformed actual mortality**	**Expected probit**	**Expected mortality (%)**	**Chi-squared statistic**	**Degrees of freedom**
10	1	90	6.28	6.00	80	2.71	8
9	0.95	75	5.67	5.88	81		
8	0.90	70	5.52	5.75	77		
7	0.85	75	5.67	5.63	74		
6	0.78	60	5.25	5.45	67		
5	0.70	50	5.00	5.25	60		
4	0.60	55	5.13	5.01	50		
3	0.48	40	4.75	4.71	39		
2	0.30	25	4.33	4.26	23		
1.5	0.18	20	4.16	3.96	15		

Chi-squared test was not significant.

**Table 2:** **Estimated 95**% **confidence interval for the LD**_**50**_**of hornet’s workers*Vespa crabro*L. and standard error (SE) of LD**_**50**_

**Species**	**LD**_**50**_**(mg/kg)** **24 h (95% CI)**	**SE of** **LD**_**50**_
*Vespa crabro* workers	4.0 mg/kg	0.39

The LD_50_ of several aculeate venoms has been determined, including toxins of different hornet species, ranging from 1.6 mg/kg for *Vespa luctuosa* Saussure venom, 2.8 mg/kg for *Vespa tropica* L., 3.1-3.8 mg/kg for *Vespa simillima* Smith, 4.1-6.1 mg/kg for *Vespa mandarinia* Smith venom, to 8.7-10.9 mg/kg for the venom of *Vespa crabro*[5,8]. However, all these values refer only to the mortality on experimental rodents, especially mice and rats. As shown by a previous study carried out on *Calliphora* sp. larvae, the toxicity of *Vespa crabro* venom is increased on these insects when compared to vertebrates, and is approximately about 2.7-7.6 mg/kg [8]. Thus, these results support the hypothesis of greater toxicity of hornet venom on its potential prey (other insects), than on mammal aggressors. Other hymenopterans have more potent venoms, such as the solitary wasp *Bracon hebetor* Say. Its LD_50_ on lepidopterous larvae was found to be less than 0.3 mg/kg [15].

Venoms of arthropods, including insects, comprise a source of numerous bioactive compounds, which evolved for prey capture and defense against predators and microorganisms. The antimicrobial, insecticidal, and hemolytic properties of peptides isolated from arthropod venoms are well known, especially concerning arachnids (scorpions and spiders) and hymenopterans (ants, wasps and bees) [15-19]. Many of these peptides have been purified and their amino acid sequences have already been characterized.

The composition and properties of the several aculeate venoms, including those of wasps and hornets, have been extensively studied [4-6,20-25]. On mammals, vespid venoms provoke prolonged pain, local edema and erythema due to increased permeability of blood vessels in the skin. Besides these direct outcomes of hornet stings, allergic reactions have also been observed in numerous cases. The generalized allergic reaction may be lethal.

In addition to their systemic effects, wasp and hornet venoms act kinetically on isolated smooth muscle and reduce blood pressure. They release endogenous histamines from granulocytes including mast cells and basophilic leucocytes; and also release catecholamines from adrenal chromaffin cells. Such toxins may also provoke cytolysis, including hemolysis and chemotaxis to macrophages and polymorphonuclear leukocytes.

The overall action of wasp and hornet venoms is complicated and may be described as an accumulation of active principles of venoms. Compounds of several wasp venoms, including venom toxins from social wasps and hornets, have been isolated and investigated. Such venoms consist of complex mixtures of active amines (serotonin, histamine, tyramine, dopamine noradrenaline and adrenaline), peptides (pain-producing peptides such as kinins, and chemotactic peptides like mastoparan or crabrolin) and proteins including many types of hydrolases (i.e. proteases, hyaluronidases, phosphatases, nucleotidases and phospholipase A), as well as allergens and neurotoxins.

Many social insects have developed defensive systems that prevent infections within their colonies. For example, bee propolis and royal jelly present antimicrobial properties and the fecal pellets of termites inhibit the development of fungal pathogens [26,27]. Concerning ants, most species possess metapleural glands on the thorax whose secretions, spread over individuals and throughout the nest, have a broad spectrum of antimicrobial action. The antibacterial property of ant venom has been demonstrated, for example, in the fire ant, whose venom alkaloids inhibit bacterial growth and presumably act as an antibiotic [28]. Venoms of honey bees, wasps and hornets, including *Vespa crabro*, possess antimicrobial peptides; however, their natural functions must be further clarified [26,29,30].

In addition to the development of social behavior, aculeate venom composition underwent evolution towards producing toxins that would be more effective against potential attackers. Usually solitary wasp venoms are employed primarily to paralyze and then kill prey [23]. Although Vespinae subfamily produces venoms that are efficient for hunting and self-defense, the most effective venom regarding defense is that of *Apis mellifera*[8,22]. Its main component is melittin, a powerful detergent that provokes hemolysis of red blood cells [30,31].

Aculeate venoms are used not only to attack prey, but also to defend the colony against foreign individuals [32]. Observations by the present author demonstrate the importance of hornet sting in nest defense against other colonies. Wasps use their venom apparatus effectively when attacking foreign workers that appear in the immediate vicinity of their nest. The toxins released in such cases are potent enough to kill (LD_50_ = 4.0 mg/kg of hornet body weight – Table 2). The determination of LD_50_ eliminates the popular myth that venomous animals can be resistant to their own venom.

The present results indicate that *Vespa crabro* venom is toxic for its own species as well as to other insects. Therefore, although both are predators, wasps and hornets as are natural allies against different pest insects and the effectiveness of their venom is proven by the relatively high values of LD_50_.

Potent venoms represent a source of new insecticidal compounds because they act selectively on their molecular targets. Such toxins affect the invertebrate nervous system and several insecticidal compounds that belong to the class of peptides or polyamine-like compounds have been purified and characterized from the venom of several hymenopterans. Numerous studies are focused on isolating and assessing the lethality of insecticidal toxins from wasps. Their venoms are expected to be used for manufacture of bioinsecticides with high selectivity for different groups of insects [29].

Animal venoms have been employed in the analysis of different physiopathological processes, and have also been involved in the design of new therapeutic drugs. Wasp toxins, due to their biological effects, may constitute potential sources of pharmacologically active compounds particularly for neuropharmacology [33]. Finally, it is worth noting that various components of venoms from wasps and bees can be used for human therapy. A classic example is the honeybee venom, which is widely employed in natural medicine (apitherapy).

## Competing interests

The author declares that there are no competing interests.
